# Arsenic speciation in freshwater fish: challenges and research needs

**DOI:** 10.1093/fqsafe/fyad032

**Published:** 2023-07-27

**Authors:** Karen S Hoy, Tetiana Davydiuk, Xiaojian Chen, Chester Lau, Jordan R M Schofield, Xiufen Lu, Jennifer A Graydon, Ruth Mitchell, Megan Reichert, X Chris Le

**Affiliations:** Department of Chemistry, University of Alberta, Edmonton, Alberta, Canada; Department of Chemistry, University of Alberta, Edmonton, Alberta, Canada; Department of Chemistry, University of Alberta, Edmonton, Alberta, Canada; Department of Chemistry, University of Alberta, Edmonton, Alberta, Canada; Department of Chemistry, University of Alberta, Edmonton, Alberta, Canada; Division of Analytical and Environmental Toxicology, Department of Laboratory Medicine and Pathology, Faculty of Medicine and Dentistry, University of Alberta, Edmonton, Alberta, Canada; Alberta Health, Health Protection Branch, Edmonton, Alberta, Canada; Alberta Health, Health Protection Branch, Edmonton, Alberta, Canada; Alberta Health, Health Protection Branch, Edmonton, Alberta, Canada; Department of Chemistry, University of Alberta, Edmonton, Alberta, Canada; Division of Analytical and Environmental Toxicology, Department of Laboratory Medicine and Pathology, Faculty of Medicine and Dentistry, University of Alberta, Edmonton, Alberta, Canada

**Keywords:** Arsenic species, environment, food consumption, freshwater fish, human exposure, identification of unknowns, liquid chromatography, mass spectrometry

## Abstract

Food and water are the main sources of human exposure to arsenic. It is important to determine arsenic species in food because the toxicities of arsenic vary greatly with its chemical speciation. Extensive research has focused on high concentrations of arsenic species in marine organisms. The concentrations of arsenic species in freshwater fish are much lower, and their determination presents analytical challenges. In this review, we summarize the current state of knowledge on arsenic speciation in freshwater fish and discuss challenges and research needs. Fish samples are typically homogenized, and arsenic species are extracted using water/methanol with the assistance of sonication and enzyme treatment. Arsenic species in the extracts are commonly separated using high-performance liquid chromatography (HPLC) and detected using inductively coupled plasma mass spectrometry (ICPMS). Electrospray ionization tandem mass spectrometry, used in combination with HPLC and ICPMS, provides complementary information for the identification and characterization of arsenic species. The methods and perspectives discussed in this review, covering sample preparation, chromatography separation, and mass spectrometry detection, are directed to arsenic speciation in freshwater fish and applicable to studies of other food items. Despite progress made in arsenic speciation analysis, a large fraction of the total arsenic in freshwater fish remains unidentified. It is challenging to identify and quantify arsenic species present in complex sample matrices at very low concentrations. Further research is needed to improve the extraction efficiency, chromatographic resolution, detection sensitivity, and characterization capability.

## Graphical Abstract

**Graphical Abstract:**
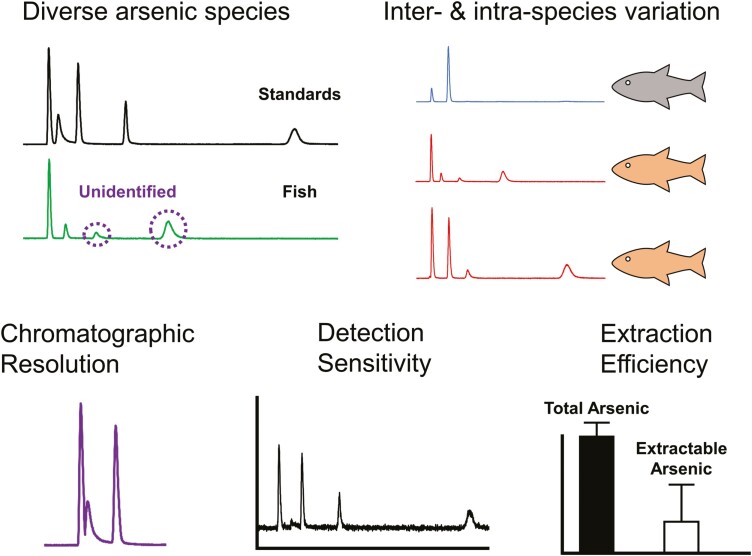

## Introduction

Food and water are the main sources of exposure to arsenic in the general population (NRC, 1999; WHO, 2018). Approximately 100–200 million people around the world are at risk of being exposed to arsenic in drinking water at a concentration higher than 10 µg/L (Uppal *et al.*, 2019; Podgorski and Berg, 2020; Zheng, 2020; He *et al.*, 2023), the World Health Organization guideline level (WHO, 2018). Chronic exposure to high concentrations of inorganic arsenic is associated with increased risks of cancers (e.g. bladder, lung, skin) and a range of other adverse health effects (e.g. diabetes, cardiovascular, neurological, developmental) (NRC, 1999; Naujokas *et al.*, 2013; WHO, 2018). However, the toxicity and chemical characteristic of arsenic varies with its chemical speciation (Styblo *et al.*, 2000; Moe *et al.*, 2016). While inorganic arsenite (As^III^) and arsenate (As^V^) are the predominant arsenic species in water, many organic arsenic species with a wide range of toxicities have been detected in the environment and biological systems (Cullen and Reimer, 1989; Francesconi, 2010; Xue *et al.*, 2022). Therefore, ­information on individual arsenic species and their identity and concentrations is important for any meaningful assessment of potential health risk associated with arsenic exposure.

Over the past seven decades, the Food and Agriculture Organization of the United Nations (FAO) has published ­global food and agriculture statistics, including the estimated consumption of various food products. The global apparent consumption of aquatic foods per capita has risen at an average annual rate of 3% from an average of 9.9 kg in the 1960s to a record high of 20.5 kg in 2019. The per-capita aquatic foods consumption in 2019 ranged from an average of 5.4 (low-income countries) to 28.1 kg (upper-middle-income ­countries). The Faroe Islands, Iceland, and the Maldives were among the countries with the largest estimated consumption of over 80 kg of aquatic foods per capita per year (FAO, 2022a).

In 2020, the worldwide production of aquatic animals was estimated at 178 million tons, with 66 million tons from inland waters (usually freshwater) and the remaining 112 million tons harvested from marine waters (FAO, 2022a). With the dominant aquatic food production being from marine sources, extensive research has focused on arsenic speciation in marine organisms. High concentrations of arsenic in marine fish and shellfish have been reported and reviewed (Francesconi, 2010; Taylor *et al.*, 2017; Xue *et al.*, 2022). However, much less is known about arsenic speciation in freshwater fish, partly because of the challenges associated with the extraction, detection, and identification of lower concentrations of arsenic species in freshwater fish. The primary objectives of this review are to (1) summarize the current state of knowledge on arsenic speciation in freshwater fish, (2) discuss the most common techniques for the determination of arsenic species, and (3) highlight remaining challenges and research needs. Understanding research needs and analytical capability will benefit future studies of arsenic speciation in freshwater fish and other food items.

## Arsenic Species in Marine Organisms

Global capture fisheries have been from 88 to 96 million tons per year since the 1990s, with the largest increase to 96.5 million tons observed in 2018 driven by an increase in marine captures. Aside from this recent increase, marine captures have remained relatively consistent since the 1980s, stabilizing at approximately 80 million tons (FAO, 2022a). Since arsenic was first reported in seafood in the 1900s, its presence in marine organisms has been researched extensively, with more than 50 arsenic species discussed in a 2010 review (Francesconi, 2010) and ‘over 300 species of naturally occurring organoarsenicals’ mentioned in a 2022 review (Xue *et al.*, 2022). Because marine seafood is an important staple in many parts of the world, ample research has been performed to quantify total arsenic and various arsenic species (LeBlanc and Jackson, 1973; Francesconi and Edmonds, 1996; De Gieter *et al.*, 2002; Kirby and Maher, 2002; Le *et al.*, 2004; Meador *et al.*, 2004; Peshut *et al.*, 2008; Hackethal *et al.*, 2021). Concentrations of arsenic in marine organisms are quite variable (Gao *et al.*, 2018; Zhang *et al.*, 2018; Ferrante *et al.*, 2019; da Silva *et al.*, 2021; Okati *et al.*, 2021; Yang *et al.*, 2021) but are generally between 5 mg/kg and 100 mg/kg (dry mass) (Francesconi, 2010). A large proportion of arsenic in marine crustaceans and fish is present as arsenobetaine—a non-toxic organoarsenic species (Bosch *et al.*, 2016; Popowich *et al.*, 2016; Thomas and Bradham, 2016; Kalantzi *et al.*, 2017). Other classes of arsenic species found in marine organisms include arsenosugars, arsenolipids, methylated and thiolated arsenicals, and As^III^ and As^V^ (Francesconi, 2010; Sele *et al.*, 2012; Khan and Francesconi, 2016; Liu *et al.*, 2021; Okati *et al.*, 2021; Xue *et al.*, 2022). Studies on arsenic speciation in marine organisms have been extensively reviewed previously. The present review focuses on arsenic speciation in freshwater fish, which has challenges in extraction, separation, quantification, and identification—partly due to lower arsenic concentrations.

## Freshwater Fish and Total Arsenic Concentration

In 2020, inland captures (12.1 million tons) and aquaculture (53.3 million tons) contributed to 37% of the global production of aquatic foods. Carps, barbels, and other cyprinids represent the main species produced for consumption (FAO, 2022a). The estimated consumption of freshwater fish, and total fish and seafood (including crustaceans, cephalopods, mollusks, and freshwater, demersal, pelagic, and marine fish) per capita of select countries in 2020 are presented in Table 1 (FAO, 2022b). The apparent consumption rates of fish (freshwater and total fish and seafood) vary among different countries; however, in general, consumption rates of freshwater fish species have been increasing worldwide.

**Table 1.** Estimated consumption of freshwater fish and total fish including seafood by population in selected countries/region in 2020 (FAO, 2022b)

Country/region	Freshwater fish (kg/capita per year)	Total fish and seafood (kg/capita per year)
Brazil	4.27	8.08
Canada	5.23	20.73
China	17.28	39.91
France	4.29	33.23
Germany	4.36	12.59
Greece	2.58	21.72
Hungary	3.12	6.35
India	6.25	7.98
Japan	5.11	46.20
Kenya	2.03	2.88
Mexico	4.32	13.63
Nigeria	1.28	6.75
Norway	10.60	50.18
Poland	1.51	12.46
Slovenia	2.47	13.21
Chinese Taiwan	6.80	29.76
Thailand	9.10	29.16
Türkiye	1.31	5.52
USA	5.34	22.79
Zimbabwe	2.57	2.97

Arsenic concentrations in freshwater fish vary as a function of many factors, including, but not limited to, their location, physiological condition, spawn status, and biological habitat, even for fish species residing in the same area (Noël *et al.*, 2013). In a study by Nevárez *et al.* (2011), it was determined that arsenic concentrations in the muscles and gills of channel catfish caught in the winter correlated with the increase of arsenic in the water and sediments. Because channel catfish are benthic omnivores with local migrations within lakes, the increase of muscle and gill arsenic may have been affected by the increase of arsenic in the sediment. In contrast, summer-caught green sunfish from the same reservoir contained the highest arsenic concentration. The seasonal arsenic differences could be related to a species habitat preference within the reservoir because the green sunfish are benthopelagic and inhabit shallow areas.

To evaluate whether aquaculture farming models could affect the arsenic concentration in fish, Ruangwises *et al.* (2012) compared the arsenic concentrations in muscle tissue of four freshwater fish species from natural water sources and aquaculture (utilizing water from natural sources). They determined the arsenic concentrations in four freshwater fish species both present in natural water sources and raised in aquaculture in Thailand. The total and inorganic arsenic concentrations in the muscle tissues were not significantly different between aquaculture-raised fish and wild-caught fish. This study suggests that routine refreshing of aquaculture waters with a water source of low arsenic concentration could reduce the concentration of arsenic in the sediment, water, and fish. Several other studies have also analyzed arsenic in freshwater fish from fisheries, aquaculture, reservoirs, urban rivers, and lakes together with nearby sediments and water sources to assess environmental impact (Nevárez *et al.*, 2011; Noël *et al.*, 2013; Szkoda *et al.*, 2014; Chua *et al.*, 2018).

In a total diet study conducted in Germany, Hackethal *et al.* (2021) analyzed a variety of fish (freshwater, marine, and migratory fish), seafood, and fish products. Freshwater fish (0.03 mg/kg) had the lowest mean total arsenic concentration, followed by migratory fish (0.73 mg/kg) and marine fish (2.82 mg/kg). Freshwater and saltwater fish residing in the Zhangzhou sea area in China’s Fujiang Province were analyzed for total arsenic. The total arsenic quantified in the 14 saltwater fish species (0.42–6.22 mg/kg, dry weight) contained arsenic 6–311 times higher than that in the two freshwater species (0.02–0.07 mg/kg, dry weight) collected in the same area (Hu *et al.*, 2023). From local markets in Shandong Province, China, Yang *et al.* (2021) collected four types of freshwater fish and six types of marine fish. The average arsenic concentration (wet weight) in the freshwater fish was 0.075 mg/kg (ranging from 0.007 to 0.500 mg/kg), much lower than that in the marine fish: an average of 1.4 mg/kg (ranging from 0.2 to 5.0 mg/kg).

Table 2 summarizes the reported concentrations of total arsenic and inorganic arsenic in freshwater fish collected from Canada, China, France, Thailand, and Türkiye (Ruangwises *et al.*, 2012; Cheng *et al.*, 2013; Noël *et al.*, 2013; Varol and Sünbül, 2018; Cui *et al.*, 2020, 2021; Tanamal *et al.*, 2021). Although freshwater fish species vary, the arsenic concentrations in freshwater fish are generally much lower than those in marine seafood in each country of study. The concentrations of individual arsenic species are even lower. The lower concentrations of arsenic in freshwater fish pose several analytical challenges for arsenic speciation analysis as discussed below.

**Table 2.** Total and inorganic arsenic in freshwater fish

Country	Collection location	Fish species	*N*	Total As (mg/kg)	Inorganic As (mg/kg)	Reference and comments
Canada	Yellowknife Bay	Lake whitefish	8	1.82±2.00	0.098±0.035	Tanamal *et al.*, 2021 Dry weight
		Northern pike	9	1.59±0.61	0.078±0.015	
		Burbot (liver)	5	4.56±2.94	0.094±0.043	
	Great Slave Lake	Lake whitefish	10	0.65±0.45	0.081±0.016	
		Northern pike	9	0.60±0.18	0.077±0.013	
		Burbot (liver)	5	1.77±0.66	0.076±0.027	
	Lower Martin Lake	Lake whitefish	10	5.97±1.46	0.050±0.025	
		Northern pike	10	3.67±0.72	0.038±0.016	
	Long Lake	Lake whitefish	10	2.65±1.49	0.061±0.009	
		Northern pike	10	3.97±1.06	0.064±0.002	
	Kam Lake	Lake whitefish	10	0.88±0.30	0.131± 0.101	
		Northern pike	10	2.36±0.92	0.077±0.018	
	Grace Lake	Lake whitefish	10	5.68±5.89	0.107±0.048	
		Northern pike	8	4.13±1.68	0.079±0.020	
	Banting Lake	Lake whitefish	10	1.50±0.76	0.087±0.023	
		Northern pike	10	2.21±0.95	0.061±0.016	
	Walsh Lake	Lake whitefish	10	1.23±0.56	0.076±0.022	
		Northern pike	10	1.54±0.55	0.077±0.018	
	Small Lake	Lake whitefish	8	0.52±0.20	0.041±0.017	
		Northern pike	8	0.42±0.11	0.044±0.020	
China	Purchased from fishery in Changsha	Crucian carp	14	0.16±0.02	—	Cui *et al.*, 2020 Dry weight
		Crucian carp (intestine)	14	0.033±0.03	—	
		Crucian carp (liver)	14	0.02±0.00	—	
		Crucian carp (kidney)	14	0.00±0.00	—	
		Crucian carp (spleen)	14	0.01±0.00	—	
	Monoculture and polyculture fish ponds around the Pearl River Delta near: Huadu, Nanhai, Shunde, Zhongshan, Jiangmen, Nansha, Dongguan, and Huizhou	Mandarin fish	27	0.13±0.04		Cheng *et al.*, 2013 Wet weight
		Northern snakehead	25	0.37±0.12		
		Largemouth bass	25	2.23±0.57		
		Grass carp	42	0.12±0.08		
		Bighead carp	15	0.20±0.09		
		Mud carp	11	0.47±0.08		
		Trash fish	9	6.76±0.59		
France	Hydrographic basins: Seine-Normandie Loire, Bretagne Rhone-Mediterranee Adour-Garonne Artois-Picardie	European eel	53	0.026–0.647		Noël *et al.*, 2013 Wet weight
		Pikeperch	7	0.029–0.233		
		Bream	19	0.029–0.331		
		Pike	6	0.017–0.356		
		Roach	57	0.027–0.255		
		Perch	2	0.034–0.039		
		Catfish	1	0.010		
		Common Carp	4	0.060–0.209		
Thailand	Chao Phraya River or Tha Chin River	Tilapia	14	0.623–1.22	0.088–0.130	Ruangwises *et al.*, 2012 Dry weight
		Silver barb	14	0.622–1.38	0.078–0.154	
		Striped catfish	15	0.573–0.965	0.072–0.126	
		Striped snakehead	14	0.924–1.89	0.188–0.414	
	Purchased from aquaculture	Tilapia	14	0.613–1.12	0.081–0.146	
		Silver barb	12	0.629–1.07	0.074–0.140	
		Striped catfish	15	0.556–1.16	0.064–0.152	
		Striped snakehead	10	0.891–2.35	0.213–0.367	
Türkiye	11 sampling sites along the Keban Dam Reservoir on Euphrates River	Euphrates barbell	44	15.3–470.8		Varol and Sünbül, 2018 Wet weight Each sample was a composite sample prepared by homogenizing 2–3 fish of same species and similar size.
		Tigris scraper	44	1.4–93.1		
		Trout barb	44	1.1–54.4		
		Common carp	44	3.3–664.8		
		Mangar	44	2.7–297.6		

Values are either mean±standard deviation (SD) or range. Unless specified, all samples were fish muscle.

## Arsenic Speciation in Freshwater Fish

Many arsenic species are present in freshwater fish. Example concentrations of the five most commonly detected arsenic species, including arsenobetaine (AsB), As^III^, As^V^, monomethylarsonic acid (MMA), and dimethylarsinic acid (DMA), are summarized in Table 3 (Šlejkovec *et al.*, 2004; Zheng and Hintelmann, 2004; Schaeffer *et al.*, 2006; de Rosemond *et al.*, 2008; Miyashita *et al.*, 2009; Jia *et al.*, 2018; Komorowicz *et al.*, 2019; Xiong *et al.*, 2020; Chen and Jiang, 2021; Hackethal *et al.*, 2021). Other previously reported arsenic species in freshwater fish include trimethylarsine oxide (TMAO), tetramethylarsonium (TETRA), arsenocholine (AsC), arsenosugars (arsenosugar-glycerol, arsenosugar-phosphate, and arsenosugar-sulfonate), arsenolipids, and ‘unknown’ arsenic species (Table 4). These ‘unknown’ arsenic species contain arsenic but their chemical structures have not been identified mainly because their low concentrations and complex sample matrix pose tremendous analytical challenges.

**Table 3.** Concentrations of five common arsenic species and other arsenic species in freshwater fish

Location and fish species	*N*	Total As (mg/kg)	As^III^^ (mg/kg)^	As^V^^ (mg/kg)^	DMA (mg/kg)	MMA (mg/kg)	AsB (mg/kg)	Concentration of other arsenic species (mg/kg)	Reference and comments
**Canada**	**Back Bay in Great Slave Lake, near Yellowknife, Northwest Territories**								
Lake whitefish	8	0.77±0.59	<0.01	<0.01	0.02±0.04	<0.08	0.08±0.09		de Rosemond *et al.*, 2008 Dry weight
Lake whitefish (liver)	8	1.07±0.58	0.07±0.08	<0.01	0.14± 0.17	<0.08	0.08±0.17		
Lake whitefish (GIT)	8	2.07±1.86	<0.01	0.01±0.01	0.10 ± 0.18	<0.08	0.01±0.04		
Walleye	8	0.57±0.19	0.05±0.08	<0.01	0.06±0.08	<0.08	0.05±0.07		
Walleye (liver)	8	1.22±0.35	0.07±0.08	0.01±0.02	0.10±0.05	<0.08	0.03±0.06		
Walleye (GIT)	7	1.48±0.31	<0.01	<0.01	<0.02	<0.08	0.02±0.04		
Northern pike	8	0.97±0.54	0.01±0.01	<0.01	0.18±0.14	<0.08	0.13±0.09		
Northern pike (liver)	8	0.42±0.13	0.07±0.08	<0.01	0.30±0.16	<0.08	0.06±0.08		
Northern pike (GIT)	8	1.82±0.61	0.16±0.32	<0.01	0.45±0.33	<0.08	0.05±0.05		
White sucker	6	0.91±0.22	0.05±0.07	0.02±0.01	0.07±0.07	<0.08	0.09±0.07		
White sucker (liver)	6	2.52±2.12	0.31±0.28	0.30±0.47	0.17±0.23	0.80±1.75	0.13±0.19		
White sucker (GIT)	6	8.92±7.51	0.10±0.15	0.12±0.18	0.02±0.02	0.49±0.74	0.04±0.05		
Longnose sucker	4	1.15±0.16	0.05±0.09	0.01±0.01	0.05±0.07	<0.08	0.12±0.10		
Longnose sucker (liver)	4	1.33±0.86	0.23±0.25	0.09±0.10	0.18±0.19	0.13±0.11	0.15±0.23		
Longnose sucker (GIT)	4	3.30±2.84	0.01±0.01	0.03±0.06	0.02±0.02	0.13±0.18	0.05±0.06		
	**Moira Lake, Ontario**								
Northern pike (*Esox lucius*)	2	0.38–0.45	0.027–0.03	0.004–0.01	0.12–0.14	N.D.–0.0005	0.06–0.08	AsC: N.D.–0.00040 TETRA: 0.016–0.042 TMAO: N.D.–0.0014 Unknown: 0.0046–0.029	Zheng and Hintelmann., 2004 Wet weight Arsenic species calculated using authors’ data. Unknown concentration is the sum of 2 unknown peaks. Unknown 1 (*t*_r: _~540 s) elutes after As^V^ in Anion Exchange Column. Unknown 2 (*t*_r: _~70 s) elutes after As^III^ as a shoulder of the As^III^ peak in Cation Exchange Column.
Largemouth bass (*Micropterus salmoides*)	1	0.20	0.03	0.02	0.02	N.D	0.03	AsC: 0.00067 TETRA: 0.043 TMAO: 0.012 Unknown: 0.028	
Yellow perch (*Perca flavescens*)	4	0.05–0.14	0.02–0.03	0.006–0.04	0.0009–0.005	0.0001–0.0008	0.0004–0.006	AsC: N.D.–0.00049 TETRA: 0.00021–0.014 TMAO: 0.00070–0.0065 Unknown: 0.00056–0.013	
Pumpkinseed (*Lepomis gibbosus*)	4	0.35–0.43	0.04–0.06	0.02–0.08	0.004–0.02	N.D.–0.006	0.01–0.02	AsC: 0.0021–0.0029 TETRA: 0.056–0.12 TMAO: 0.014–0.023 Unknown: 0.0083–0.030	
**China**	**Locations include: Changsha, Hengyang, Xiangtan, Yongzhou, Yueyang, and Zhuzhou**								
White amur bream (*Parabramis pekinensis*)	16	0.15±0.03–1.03±0.34	0.01±0.02–0.06±0.04	0.04±0.02–0.17±0.13	N.D.–0.09±0.09	N.D.– 0.01±0.01	0.05±0.01–0.40±0.06	N.D.–0.015±0.018	Jia *et al.*, 2018 Dry weight Other arsenic species=AsC. *No reported standard deviation.
Barbel chub (*Squaliobarbus curriculus*)	22	0.69±0.26–1.39±0.25	0.004±0.001–0.10±0.08	0.04±0.03–0.17±0.14	0.05±0.01–0.27±0.11	N.D.–0.02±0.01	0.23±0.03–0.51±0.23	N.D.–0.005±0.002	
Crucian carp (*Carassius auratus*)	23	0.19±0.01–0.75±0.30	0.01±0.001–0.02±0.02	0.03±0.03–0.06±0.04	0.02±0.01–0.17±0.18	N.D.–0.02±0.02	0.08±0.03–0.38±0.15	N.D.–0.024±0.016	
Common carp (*Cyprinus carpio*)	7	0.53±0.36–0.80±0.19	0.02±0.01–0.14±0.05	0.05±0.06–0.11±0.14	N.D.–0.20±0.23	N.D.–0.01*	0.24±0.15–0.34±0.22	N.D.–0.060±0.023	
Yellow catfish (*Pelteobagrus fulvidraco*)	22	0.33±0.06–2.84±1.05	0.01±0.001–0.12±0.13	0.04±0.05–0.19±0.11	N.D.–0.07±0.05	N.D.–0.01*	0.14±0.05–1.86±0.48	N.D.–0.072±0.060	
Amur catfish (*Silurus asotus*)	20	0.06±0.01–2.03±0.77	0.01±0.002–0.06±0.05	0.01±0.003–0.11±0.05	N.D.–0.04±0.004	N.D.–0.01*	0.03±0.47–1.29±0.01	N.D.–0.014±0.011	
Mandarin fish (*Siniperca chuatsi*)	6	0.39±0.06–1.61±0.87	0.02±0.02–0.11±0.07	0.01±0.01–0.29±0.34	0.05±0.01–0.18±0.07	N.D.–0.05*	0.06±0.04–0.16±0.03	0.004±0.001–0.019±0.001	
Stone moroko (*Pseudorasbora parva*)	4	0.30±0.08–0.51±0.13	0.004±0.002–0.01±0.01	0.03±0.02–0.06±0.02	0.03±0.01–0.03±0.01	N.D.	0.17±0.01–0.35±0.20	0.003±0.001–0.005±0.001	
**Germany**	**Locations include: Berlin, North, East, South or West Germany**								
Striped catfish	20*	0.01	iAs (0.006)**	–	N.D.	N.D.	0.01	–	Hackethal *et al.*, 2021 Dry weight *Denotes number of individual samples collected and pooled into 1 sample. **Concentration of all inorganic arsenic species.
Carp (region 1)	15*	0.05	–	–	N.D.	0.03	0.01	–	
Carp (region 2)	15*	0.03	–	–	N.D.	0.01	0.02	–	
Carp (region 3)	15*	0.03	–	–	N.D.	0.02	0.01	–	
Carp (region 4)	15*	0.06	–	–	N.D.	0.03	0.02	–	
**Hungary**	**River Danube at City of Paks**								
Fry (mixed fish) days old	1	1.37	<0.02	0.1	<0.03	<0.03	<0.02	AsC: <0.02 AsSugar-OH: Trace AsSugar-PO_4_: 0.12 AsSugar-SO_3_^–^: <0.08 TETRA: <0.02 Thio-AsSugar-OH: <0.04 Thio-AsSugar-PO_4_: <0.04	Schaeffer *et al.*, 2006 Dry weight As^III^ values are estimated. Arsenic speciation performed only on samples with>0.5 mg/kg of total arsenic.
Fry (mixed fish) months old	1	1.21	<0.02	Trace	<0.03	<0.03	<0.02	AsC: <0.02 AsSugar-OH: <0.02 AsSugar-PO_4_: 0.12 AsSugar-SO_3_^–^: <0.08 TETRA: <0.02 Thio-AsSugar-OH: <0.04 Thio- AsSugar-PO_4_: <0.04	
Silver carp	1	1.17	<0.02	<0.03	<0.03	<0.03	<0.02	AsC: <0.02 AsSugar-OH: <0.02 AsSugar-PO_4_: <0.03 AsSugar-SO_3_^–^: <0.08 TETRA: <0.02 Thio-AsSugar-OH: <0.04 Thio-AsSugar-PO_4_: <0.04	
White bream A	1	1.58	<0.02	<0.03	<0.03	<0.03	Trace	AsC: <0.02 AsSugar-OH: Trace AsSugar-PO_4_: 0.24 AsSugar-SO_3_^–^: <0.08 TETRA: <0.02 Thio-AsSugar-OH: <0.04 Thio-AsSugar-PO_4_: 0.07	
White bream B	1	0.71	<0.02	<0.03	<0.03	<0.03	0.03	AsC: <0.02 AsSugar-OH: <0.02 AsSugar-PO_4_: Trace AsSugar-SO_3_^–^: <0.08 TETRA: <0.02 Thio-AsSugar-OH: <0.04 Thio-AsSugar-PO_4_: 0.07	
**Japan**	**Hayakawa River in Kanagawa**								
*Plecoglossus altivelis*	2*	0.86±0.41	N.D.	0.01±0.01	0.03±0.04	N.D.	0.12±0.09	AsSugar-PO_4_: 0.0081±0.011 TMAO: 0.064±0.09	Miyashita *et al.*, 2009 Dry weight *Analytical results for at least 2 samples. LOQ=0.00025
*Anguilla japonica*	2*	0.15±0.02	N.D.	0.0003±0.00	0.03±0.006	0.0003±0.00	0.008±0.003	AsSugar-OH: <0.00025 AsSugar-PO_4_: <0.00025	
*Cobitis biwae*	1	0.66	N.D.	<0.00025	<0.00025	0.02	0.14	AsSugar-PO_4_: 0.036	
*Leuciscus hakonensis*	2*	0.30±0.03	N.D.	0.006±0.008	0.01±0.007	N.D.	0.02±0.01	AsSugar-OH: 0.015±0.0074 TMAO: 0.011±0.0030	
*Phoxinus lagowski steindachneri*	2*	0.31±0.14	N.D.	0.02±0.03	0.01±0.02	<0.00025	0.05±0.01	AsSugar-OH: 0.0020±0.0036 AsSugar-PO_4_: 0.0041±0.0079 TMA: 0.023±0.020 TMAO: 0.022±0.027	
*Rhinogobius* sp*. CB*	2*	0.72±0.12	N.D.	0.006±0.006	0.003±0.003	<0.00025	0.10±0.03	AsC: 0.0049±0.0048 AsSugar-OH: 0.0085±0.014 AsSugar-PO_4_: 0.024±0.010 TMAO: 0.0011±0.0022	
*Rhinogobius* sp*. CO*	2*	0.70 ± 0.12	N.D.	0.001±0.002	0.001±0.002	<0.00025	0.17±0.08	AsC: 0.0056±0.0074 AsSugar-OH: 0.0030±0.0041 AsSugar-PO_4_: 0.028±0.020 TMAO: <0.00025 Unknown: 0.0020± 0.0044	
*Sicyopterus japonicus*	2*	2.10±0.49	N.D.	0.0051±0.0046	0.04±0.03	N.D.	0.29±0.05	AsSugar-PO_4_: 0.078±0.023 TMAO: 0.018±0.017	
*Zacco platypus*	2*	1.00±0.50	N.D.	0.0003±0.00	0.04±0.03	N.D.	0.03±0.01	AsSugar-OH: 0.0073±0.010 AsSugar-PO_4_: 0.089±0.021 TMAO: 0.013±0.019	
**Norway**	**Purchased from aquaculture in Graz**								
Steamed salmon	1	2.63±0.03	–	–	0.017±0.001	–	1.72±0.05	AsC: 0.001±0.0002 AsHC 332+AsHC 404: 0.0217±0.002 AsHC 360: 0.012±0.002 Less polar lipid-soluble 1: 0.009±0.0003 Less polar lipid-soluble 2: 0.018±0.0017 TETRA: 0.0007±0.0002 TMAO: 0.002±0.0003	Xiong *et al.*, 2020 Dry weight
**Poland**	**Collected from Wielkopolska or Lower Silesia**								
Silver bream 1	1	0.09	–	N.D.	–	–	0.08	–	Komorowicz *et al.*, 2019 Dry weight
Silver bream 2	1	0.12	–	N.D.	–	–	0.09	–	
Bream	1	0.52	–	N.D.	–	–	0.45	–	
Carp	1	0.07	–	N.D.	–	–	0.06	–	
Bream	1	0.38	–	0.01	–	–	0.30	–	
Trout 1	1	4.52	–	0.13	–	–	3.87	–	
Trout 2	1	4.82	–	0.06	–	–	4.16	–	
Sturgeon	1	5.93	–	0.04	–	–	5.23	–	
**Slovenia**	**Locations collected from include: Drava, Idrijca, Krka, Ljubljanica, Meža, Nadiža, Titanava mlinščica, Savinja, Sava, Sora, and Soča or cultivated**								
Catfish	1	0.81	N.D.	–	N.D.	–	0.004±0.0009		Šlejkovec *et al.*, 2004 Wet weight Traces=clearly present at concentrations just above detection limit of 0.0015 mg/kg. Unknown (*t*_r_=375 s) on Cation Exchange Column. Concentrations estimated using TMAO peak areas.
Burbot	1	0.10	N.D.	–	0.057±0.005	–	0.007±0.003	Unknown: 0.011	
Barbel	3	0.09	N.D.	–	N.D.	–	0.007±0.0003	Unknown: 0.010	
Danube roach 1	2	0.09	N.D.	–	N.D.	–	0.017±0.001	Unknown: 0.015	
Danube roach 2	1	0.12	Traces	–	N.D.	–	0.022±0.003	TMAO: 0.0044 Unknown: 0.005	
Nase 1	10	0.10	N.D.	–	0.002±0.0002	–	N.D.	TMAO: 0.0015 Unknown: 0.004	
Nase 2	2	0.14	N.D.	–	0.004±0.0007	–	0.002±0.0006	Unknown: 0.004	
Nase 3	1	0.25	Traces	–	0.005±0.0006	–	N.D.	–	
Marble trout	3	0.87	N.D.	–	N.D.	–	0.78±0.01	–	
Rainbow trout 1	1	0.53	N.D.	–	N.D.	–	0.42±0.03		
Rainbow trout 2	1	0.15	Traces	–	N.D.	–	0.11±0.01	–	
Brown trout 1	3	0.08	Traces	–	N.D.	–	0.047±0.004	–	
Brown trout 2	3	0.08	0.005±0.0004	–	N.D.	–	0.055±0.003	–	
Brown trout 3	6	0.62	N.D.	–	0.03±0.003	–	0.40±0.04	–	
Brown trout 4	4	1.24	N.D.	–	0.04±0.0005	–	0.66±0.03	–	
Inbreed between marble and brown trout	1	0.61	N.D.	–	N.D.	–	0.82±0.02	–	
**Chinese Taiwan**	**Purchased locally**								
Freshwater tilapia	1	0.38	0.04	0.05	0.07	0.02	0.19	–	Chen and Jiang., 2021 Dry weight
Freshwater bass	1	0.26	0.03	0.05	0.03	0.02	0.12	–	

Unless specified, all samples were fish muscle. Values reported as mean±standard deviation and/or range (–). N.D.=not detectable, GIT=gastrointestinal tract.

**Table 4.** Arsenic species detected in freshwater fish and discussed in this review

Arsenic species (abbreviation)	Structure
**Arsenobetaine (AsB)**	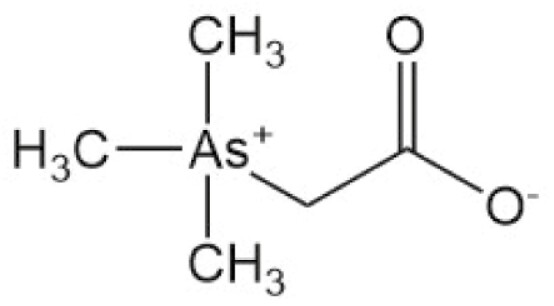
**Arsenocholine (AsC)**	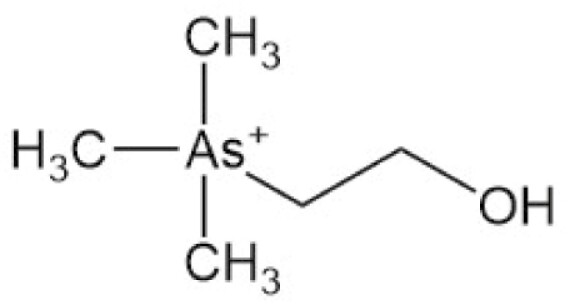
**Inorganic arsenite (As** ^**III**^)	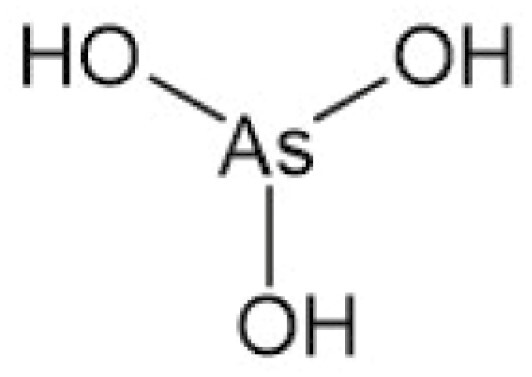
**Inorganic arsenate (As** ^**V**^)	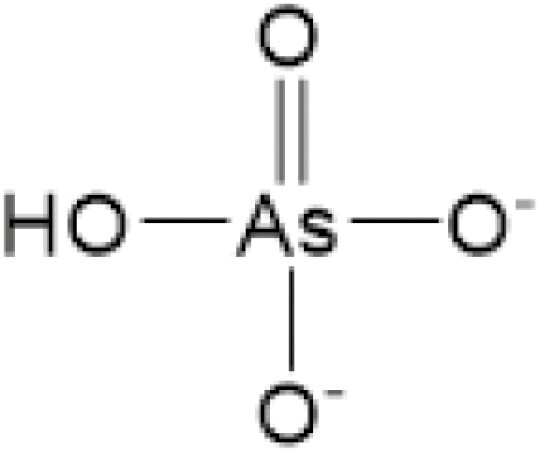
**Monomethylarsonic acid (MMA)**	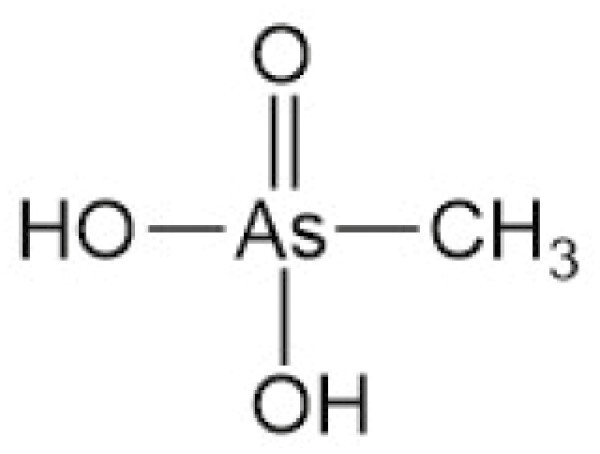
**Dimethylarsinic acid (DMA)**	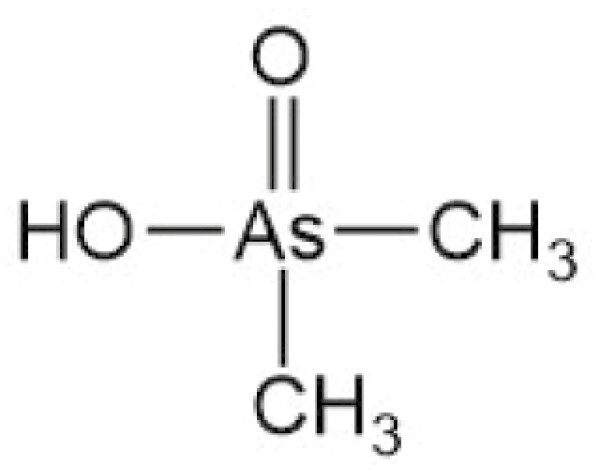
**Trimethylarsine oxide (TMAO)**	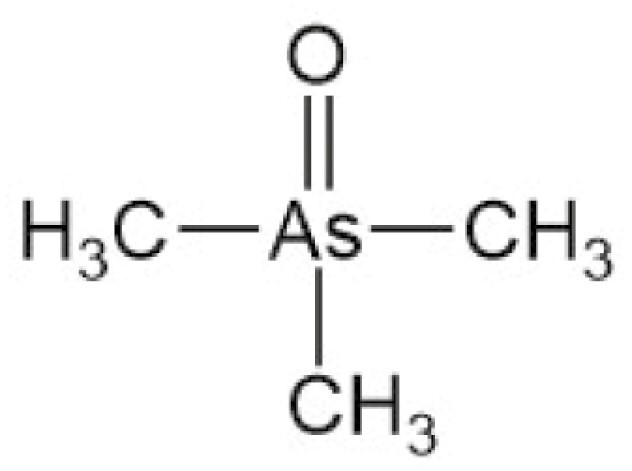
**Tetramethylarsonium (TETRA)**	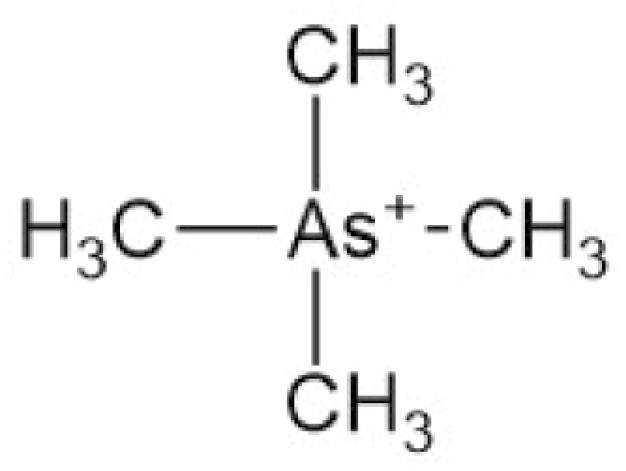
**Arsenosugars (AsSugars)**	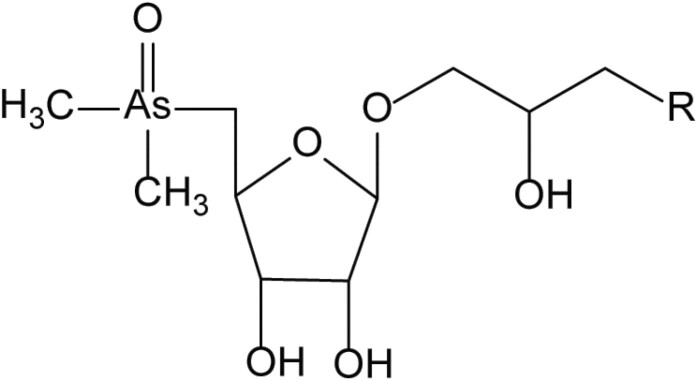
Arsenosugar-glycerol (AsSugar-OH) R=OH	
Arsenosugar-sulfonate (AsSugar-SO_3_) R=SO_3_^–^	
Arsenosugar-phosphate (AsSugar-PO_4_) R=	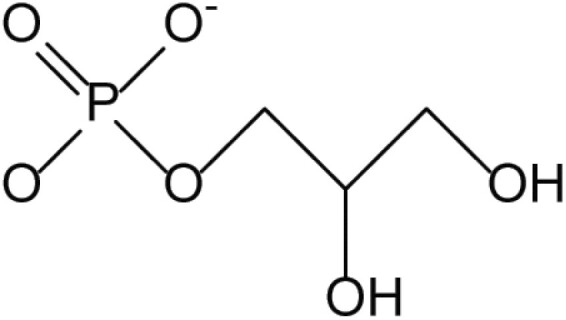
**Thiolated arsenosugars (Thio-AsSugars)**	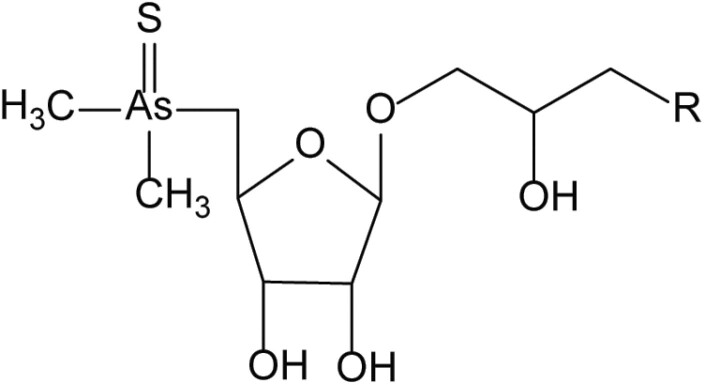
**Arsenolipids**	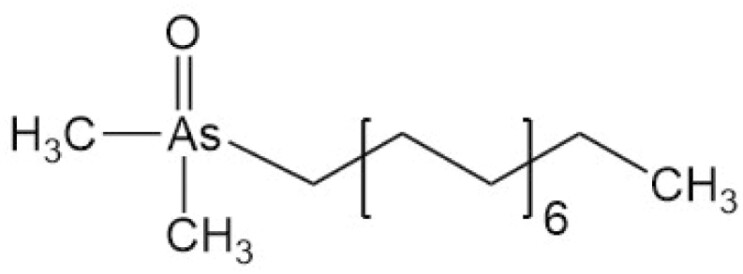
Arsenic-containing hydrocarbon	
AsHC (332)	
Arsenic-containing fatty acid	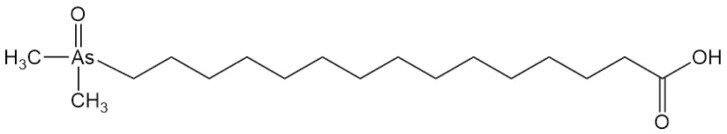
AsFA (362)	
Arsenic-containing phosphatidylcholine	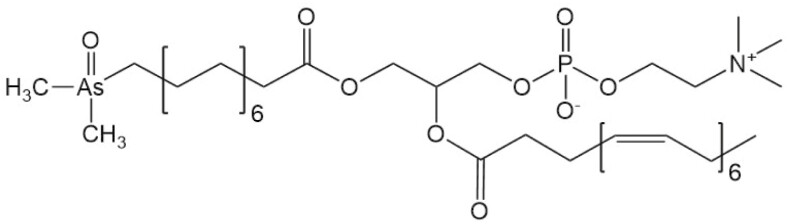
AsPC (911)	

### Arsenic speciation patterns in fish muscle

Concentrations of AsB, As^III^, As^V^, MMA, DMA, and ‘unknown’ arsenic species were determined by Tanamal *et al.* (2021) in northern pike and lake whitefish muscle tissue. The dominant arsenic species in both fish species was AsB. DMA concentrations were significantly higher in northern pike than in lake whitefish samples. Inorganic arsenic proportions varied within the same fish species collected from different lakes, with a greater total inorganic arsenic proportion in lake whitefish than in northern pike.

Determination of total arsenic, MMA, DMA, As^III^, and As^V^ in fish muscle at different trophic levels from two lakes has also been investigated (Yang *et al.*, 2017). The proportion of arsenic species (inorganic arsenic, MMA, and DMA) in the herbivorous, omnivorous, and carnivorous fish was not consistent between the two lakes. This variability in arsenic speciation was observed for fishes at the same trophic level, but also for the same fish species from different lakes.

To investigate whether arsenic speciation patterns are unique to freshwater fish at the family or species level, Šlejkovec *et al.* (2004) determined arsenic compounds present in the fish muscle from the Cyprinidae, Gadidae, and Salmonidae families. AsB was detected in most samples, with DMA and AsB as the dominant species in the Gadidae and Salmonidae families, respectively. In the Cyprinidae family, DMA was detected in all three nase samples, while AsB and ‘unknown’ arsenic species were the dominant arsenic compounds extracted in the barbel and Danube roach fish. This study, along with many others presented here and in Table 3, suggests that fish metabolism, diet, and environment can affect the arsenic speciation in muscles.

### Arsenic distribution within a fish

Several arsenic speciation studies have focused on the distribution of arsenic in muscles (Charette *et al.*, 2021) and other organs, e.g. liver, kidney, gill, gastrointestinal tract (GIT), skin, spleen, and eggs (Maher *et al.*, 1999; de Rosemond *et al.*, 2008; Harkabusová *et al.*, 2009; Yang *et al.*, 2017; Pei *et al.*, 2019; Tanamal *et al.*, 2021). Charette *et al.* (2021) compared the intramuscular arsenic distribution between three muscle types (white, intermediate, and red). Striped bass possess all three muscle types, in contrast to the northern pike, which only has white muscles. The authors observed a positive correlation between arsenic species and the higher lipid content of red muscles in striped bass. The higher lipid content (2.3 times) red muscle contained 265% more arsenolipids and 57% less AsB compared to the white muscle.

The distribution of arsenic varies between different fish organs and tissues. Yang *et al.* (2017) reported relative average arsenic concentrations in the organs of 10 freshwater fish in decreasing concentration as follows: liver>gill>muscle>skin>eggs. de Rosemond *et al.* (2008) investigated arsenic species in muscle, liver, and GIT of five different fish species (lake whitefish, walleye, northern pike, white sucker, and longnose sucker). AsB and DMA were found in the muscle, liver, and GIT of all the fish species. The predominant arsenic species in the liver of the northern pike was DMA. As^III^ was detected in all livers and GIT of northern pike, white sucker, and longnose. The arsenic distribution in all fish was GIT>liver>muscle, except for the northern pike, in which the distribution was GIT>muscle>liver. In a tilapia exposure study, the total arsenic concentration was reported in the following decreasing order: GIT>liver>gill>muscle (Pei *et al.*, 2019). However, the distribution of arsenic species varied in tilapia, where the highest proportion of organic arsenic was in the muscle (90%)>gills>liver>GIT. AsB, As^III^, As^V^, MMA, and DMA were found in all tissues when fish were exposed to As^III^ and As^V^. AsB was the major species constituting up to 75% of the total arsenic in gill tissue and DMA was the second predominant species in all tissues.

### Arsenic species not commonly reported or not identified

**Figure 1.**
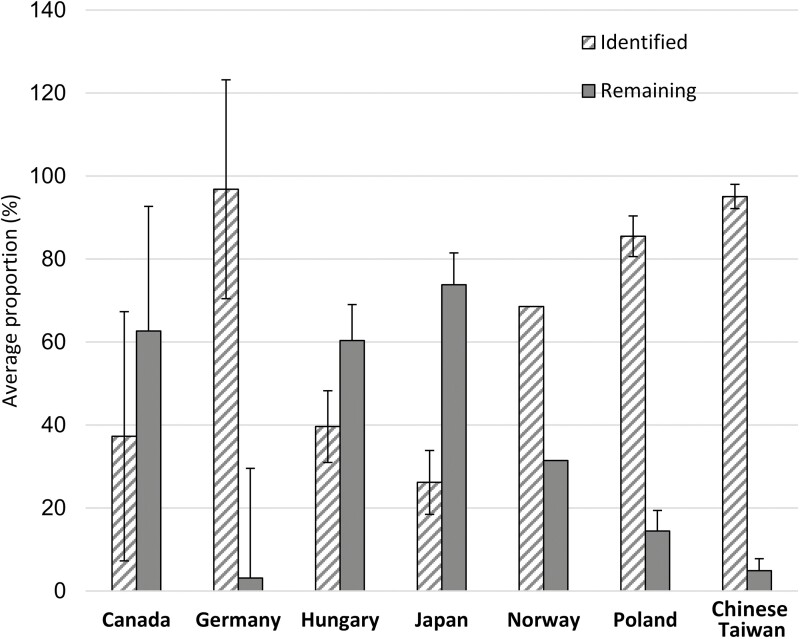
Average proportion of identified arsenic species and other arsenic species remaining unidentified in freshwater fish from selected areas. Proportions (%) were calculated using the mean of the sum of identified arsenic species and the total arsenic determined for fish species in each study. Error bars represent the standard deviation. Values for the concentration of total arsenic and arsenic species can be found in Table 3. In the Norway study, only one sample was reported. (References: Schaeffer *et al.*, 2006; de Rosemond *et al.*, 2008; Miyashita *et al.*, 2009; Komorowicz *et al.*, 2019; Xiong *et al.*, 2020; Chen and Jiang, 2021; Hackethal *et al.*, 2021).

The main challenge of arsenic speciation in freshwater fish is the presence of many arsenic species that remain unidentified. Although the five most commonly reported arsenic species in fish are AsB, As^III^, As^V^, MMA, and DMA (Table 3), the sum of their concentrations is often much lower than the total arsenic concentration. Studies conducted in Canada (de Rosemond *et al.*, 2008), Hungary (Schaeffer *et al.*, 2006), Japan (Miyashita *et al.*, 2009), and Norway (Xiong *et al.*, 2020) have shown that 31%–74% of the total arsenic in fish was not accounted for by the sum of all identified arsenic species (Figure 1). Some of this remaining 31%–74% arsenic might be those not fully extracted from fish tissue, and others might be unidentified, unknown arsenic species. The ‘unknown’ arsenic species can make up a few percent to more than half of the total arsenic. Without knowing what arsenic species make up the difference between the total arsenic and the five arsenic species commonly detected, it is difficult to provide any meaningful assessment of exposure and health risk. Because the toxicity and biochemical behavior of arsenic vary with the chemical species of arsenic, it is important to identify unknown arsenic species and quantify all arsenic species. The identification and quantification of arsenic species require efficient extraction, chromatographic separation, and complementary detection, which are discussed in the following sections.

## Sample Preparation and Extraction of Arsenic Species

Preparation of samples and extraction of arsenic species are critical components for arsenic speciation analysis. The integrity of arsenic species and their concentrations must be maintained throughout the sample preparation and extraction processes, without losses, contamination, or interconversion of arsenic species (B’Hymer and Caruso, 2004). When various arsenic species are present at very low concentrations, such as in the case of freshwater fish, it is very challenging to extract and quantify individual arsenic species.

Fish muscle has been commonly analyzed for the assessment of human consumption, although the liver, kidneys, gills, spleen, skin, and eggs have also been analyzed, most often for studies of arsenic distribution in different organs (Šlejkovec *et al.*, 1996; Maher *et al.*, 1999; Zheng and Hintelmann, 2004; Schaeffer *et al.*, 2006; de Rosemond *et al.*, 2008; Harkabusová *et al.*, 2009; Arroyo-Abad *et al.*, 2016; Juncos *et al.*, 2019). Alava *et al.* (2012) stressed that particle size was an important factor in the extraction efficiency of arsenic species; therefore, fish tissues were either ground (wet weight) or freeze-dried for subsequent analysis. Freeze-dried samples were usually homogenized to very fine particles using a clean mortar and pestle (Zwicker *et al.*, 2011), agate ball mill (Shah *et al.*, 2010), blade grinder (Zhao *et al.*, 2018) or bullet processor (Tanamal *et al.*, 2021).

The most common solvents for arsenic extraction are methanol and water mixtures in different ratios. The extraction process was usually enhanced using a sonication water bath (Šlejkovec *et al.*, 1996; Zheng and Hintelmann, 2004; Liu *et al.*, 2014; Arroyo-Abad *et al.*, 2016; Yang *et al.*, 2017; Komorowicz *et al.*, 2019), sonication probe (Šlejkovec *et al.*, 2004; Batista *et al.*, 2012; Komorowicz *et al.*, 2019), shaking top over bottom (Soeroes *et al.*, 2005a, 2005b; Schaeffer *et al.*, 2006), and microwave heating (Reyes *et al.*, 2009; Ruttens *et al.*, 2012; Komorowicz *et al.*, 2019; Wang *et al.*, 2019). Pizarro *et al.* (2003) studied arsenic speciation in environmental and biological samples (e.g. fish, rice, and chicken) using water and methanol:water extraction under sonication. They concluded that the best extraction efficiency (96%) was achieved using methanol:water (1:1). Zwicker *et al.* (2011) compared extractions using water, methanol:water (1:1), and methanol:water (1:9), all under sonication for 1 h. They found that both water and the methanol:water (1:9) mixture yielded the highest extraction efficiency of arsenic from two reference materials: DORM-2 (95% and 94%, respectively) and TORT-2 (81% and 82%, respectively). Ciardullo *et al.* (2010) studied the extraction of arsenic species from eel, chub, mullet, and carp using methanol:water (1:1). They observed that repeated extraction (three times) was better than a single extraction for eel and mullet, and that a larger volume of methanol:water extracted more arsenic species from chub but not from carp. The combined extracts (three times) were evaporated to almost dryness to remove excess solvents, and then the extracts were dissolved in deionized water for subsequent analysis.

Organic solvents, such as acetone, chloroform, dichloromethane (DCM), hexane, methyl-*tert*-butylether (MTBE), and pyridine (Maher *et al.*, 1999; McKiernan *et al.*, 1999; de Rosemond *et al.*, 2008; Stiboller *et al.*, 2019), have been used to complement the extraction by water and methanol. Amayo *et al.* (2014) extracted arsenic species sequentially: first, 20 mL hexane was mixed with fish muscle, shaken overnight at room temperature, and centrifuged; then, the residue was further extracted with methanol:DCM (1:2). Pereira *et al.* (2016) sequentially extracted arsenic species from marine fish oil using hexane, followed by methanol:chloroform (1:2), methanol, and water. Charette *et al.* (2021) used chloroform:methanol (2:1) for the first ­extraction, and then chloroform:methanol:water (2:1:1) for the second extraction of arsenic species from striped bass and northern pike. Most of these studies focused on the extraction of arsenolipids using chloroform/DCM/hexane, and the methanol–water extracts contained water-soluble arsenic species.

A mixture of 1%–2% diluted HNO_3_ with 1% H_2_O_2_ or 10% methanol has also been used for the extraction of arsenic species from freshwater fish (Batista *et al.*, 2012; Juncos *et al.*, 2019). Zhao *et al.* (2018) used water, 10% H_3_PO_4_, 3% acetic acid, and 10% HNO_3_, to examine the extraction efficiency of 11 arsenic species: AsB, DMA, As^III^, AsC, MMA, As^V^, and five phenylarsenicals. They found that water and aqueous acidic solutions were not satisfactory for the extraction of all arsenic species. When using water as the solvent, the recovery of As^III^ was 23.9%, while that of other species was 90%−96%. With 10% H_3_PO_4_ as the solvent, only AsB and AsC had recovery over 90%, while the recoveries of other arsenic species were 52%−87%. A similarly low recovery was observed with the extraction solvents 3% acetic acid (43%−89% recovery) and 10% HNO_3_ (19.8%−79.9%).

It has been suspected that the low extraction efficiency of inorganic arsenic from biological matrices could be due to the inability of the water–methanol mixture to break the bonds between As^III^ and thiol groups in proteins (Reyes *et al.*, 2009). Various enzymes, such as trypsin, pancreatin, pepsin, proteinase K, pronase E/lipase, protease XIV/α-amylase, protease, cellulase, viscozyme, and a combination of enzymes, have been used to assist the extraction of arsenic species from fish and plant samples (Viňas *et al.*, 2003; Sanz *et al.*, 2005; Guzmán Mar *et al.*, 2009; Reyes *et al.*, 2009; Dufailly *et al.*, 2011; Moreda-Piñeiro *et al.*, 2011; Wolle *et al.*, 2014; Sadee *et al.*, 2016; Wolle and Conklin, 2018; Zhao *et al.*, 2018). The functions of these enzymes were to help digest fats, break cell walls, and hydrolyze peptide bonds in proteins. Enzyme-assisted extraction was typically conducted in a buffer ­solution, e.g. Tris–HCl or phosphate buffer, that is compatible with the selected enzymes. Viňas *et al.* (2003) used 100 mg trypsin or pancreatin in 15 mL 0.1 mol/L NH_4_HCO_3_ solution. The mixture of sample and enzymes was homogenized using a mortar and pestle to grind the mixture for 3 min, and then the mixture was placed in a shaking bath at 37 °C overnight. They achieved 100% extraction efficiency of AsB from trout fish and the matrix effect was negligible. Zhao *et al.* (2018) evaluated three enzymes used for the extraction of arsenic species from marine and freshwater fish. They analyzed silver carp and Chinese sturgeon fish and detected AsB, AsC, DMA, As^III^, and As^V^. Sanz *et al.* (2005) mentioned that protease type XIV and α-amylase mixture solution itself contained ­background arsenic (up to 0.2% for As^V^). Therefore, purification of enzyme solution was necessary to minimize the ‘reagent blank’ of the extraction methods (Dufailly *et al.*, 2011).

Another issue of the enzyme-assisted extraction is the ­extensive incubation time (several hours) often required for enzymatic hydrolysis. To speed up the extraction, researchers combined enzyme-assisted with microwave-assisted (or sonication) approaches to extract arsenic species from seafood, seaweed, staple diets (fish and rice), baby food, and plants (Guzmán Mar *et al.*, 2009; Reyes *et al.*, 2009; Dufailly *et al.*, 2011; Moreda-Piñeiro *et al.*, 2011; Wolle *et al.*, 2014; Sadee *et al.*, 2016; Wolle and Conklin, 2018). Reyes *et al.* (2009) optimized microwave-assisted extraction procedures based on the amounts of pronase E and lipase, pH, and microwave irradiation time. They chose 20 mg pronase E, 5 mg lipase, 10 mL of 50 mmol/L phosphate buffer (pH 7.25), and 30 min for extraction of 200 mg DOLT-3 certified reference material (CRM). Dufailly *et al.* (2011) optimized the extraction conditions as sonication time 5 min, sonication power (60%), and 3 mL of 10 mg/mL protease XIV and applied the method to the extraction of arsenic species from SRM 1568a (rice flour) and CRM 627 (tuna fish). The reported recoveries were based on the arsenic species spiked in to rice (97%−122%), baby food (95%−111%), and tuna fish (93%−114%). Chen and Jiang (2021) optimized various ­extraction solutions and selected a mixture of 1% (volume fraction) HCl and 0.1% (mass concentration) protease XIV, along with microwave-assisted extraction. They detected AsB, As^III^, As^V^, MMA, and DMA in freshwater tilapia and bass and reported an extraction efficiency of more than 95%. Most of these studies did not examine any possible conversion of arsenic species during the extensive extraction processes.

The stability of arsenic species during sample preparation can be affected by factors such as extraction solutions, sonication or microwave energy, and temperature. For example, alkaline tetramethylammonium hydroxide (TMAH) dissolves the sample matrix and efficiently releases arsenic species (Serafimovski *et al.*, 2006; Wolle and Conklin, 2018); however, TMAH has been observed to convert arsenic species. Wolle and Conklin (2018) reported 92%−94% recovery of the total arsenic when 5% TMAH was used to extract arsenic species from seafood, seaweed, and CRMs. However, As^III^ was oxidized to As^V^, and arsenosugars with a mass-to-charge ratio (*m*/*z*) of 392, 408, and 482 were converted to the arsenosugar 328 (*m*/*z*). Similarly, Luvonga *et al.* (2021) compared 0.2 mol/L HNO_3_ and 6% H_2_O_2_ to the water extraction method. Although there was no difference in AsB, AsC, MMA, DMA, TMAO, and As^V^ between water extraction and acid extraction, As^III^ was completely converted to As^V^ under acid extraction. Arsenosugars with –OH, –SO_3_, and –PO_4_ moieties were converted to arsenosugar 254 under acid extraction.

Extracted arsenic species and their extraction efficiency vary among fish species. Luvonga *et al.* (2021) found that the extraction efficiency for wild-caught salmon (54%) was significantly lower than that for aquacultured salmon (82%). The difference in extraction efficiency between wild-caught salmon and aquaculture salmon might be attributed to a more non-extractable fatty acid portion in wild-caught salmon. These results suggest that optimization of extraction methods is required for different fish species. Table 5 summarizes the common arsenic species extracted and extraction efficiencies using a variety of extraction methods. The variabilities in extraction efficiencies among the reported studies reflect biological differences in fish samples but also indicate challenges in extracting arsenic species from freshwater fish for speciation analysis.

**Table 5.** Summary of extraction procedures, arsenic species extracted, and extraction efficiencies of various freshwater fish.

Extraction procedure	Fish species	As species	Extraction efficiency (%)	Reference
Methanol:water 1:1, mechanical agitation, 15 h overnight, three times extraction, and evaporated	Eel	As^III^, As^V^, MMA, DMA, **AsB**, TMAO, AsC, TETRA, AsSugar-OH, Thio-AsSugar-PO_4_, unknown 1	81–84	Ciardullo *et al.*, 2010
	Mullet	As^III^, DMA, **AsB**, TMAO, AsC, TETRA, AsSugar-OH, Thio-AsSugar-PO_4_, unknown 1	79–82	
	Chub	As^III^, DMA, **AsB**, TMAO, AsC, TETRA, AsSugar-OH, AsSugar-SO_4_, Thio-AsSugar-PO_4_, unknown 1, unknown 2	81–89	
	Carp	As^III^, As^V^, DMA, **AsB**, TMAO, AsC, TETRA, AsSugar-OH, Thio-AsSugar-PO_4_, AsSugar-PO_4_, unknown 1	62–64	
Methanol:water 1:1, standing for 10 min, sonicated for 10 min, and centrifuged for 2 min at 5200 r/min	*Plecoglossus altivelis*	As^V^, DMA, **AsB**, TMAO, AsSugar-PO_4_	28	Miyashita *et al.*, 2009
	*Anguilla japonica*	As^V^, MMA, **DMA**, AsB, AsSugar-OH, AsSugar-PO_4_	25	
	*Cobitis bjwae*	As^V^, MMA, DMA, **AsB**, AsSugar-PO_4_	31	
	*Leuciscus hakonesis*	As^V^, DMA, **AsB**, TMAO, AsSugar-OH	21	
	*Phoxinus lagowski steindachneri*	As^V^, MMA, DMA, **AsB**, TMAO, TETRA, AsSugar-OH, AsSugar-PO_4_	42	
	*Rhinogobius* sp. CB	As^V^, MMA, DMA, **AsB**, TMAO, AsC, AsSugar-OH, AsSugar-PO_4_	21	
	*Rhinogobius* sp. CO	As^V^, MMA, DMA, **AsB**, TMAO, AsC, AsSugar-OH, AsSugar-PO_4_, unknown	30	
	*Sicyoterus japonicas*	As^V^, DMA, **AsB**, TMAO, AsC, AsSugar-PO_4_	20	
	*Zacco platypus*	As^V^, DMA, AsB, TMAO, AsSugar-OH, **AsSugar-PO**_**4**_	17	
Pure water, sonicated 30 s using ultrasonic probe, shaken top over bottom overnight, and centrifuged for 15 min at 4500 r/min	Fry, days old	As^V^, **AsSugar-PO**_**4**_	26	Schaeffer *et al.*, 2006
	Fry, months old	TMAO, **AsSugar-PO**_**4**_	27	
	Sliver carp	**TMAO**	12	
	White bream A	**AsSugar-PO**_**4**_, Thio-AsSugar-PO_4_	59	
	White bream B	AsB, **Thio-AsSugar-PO**_**4**_	36	
Methanol:water 1:1 mixed with omni-mixer, sonicated for 20 min, and centrifuged for 15 min at 5000 r/min	Northern pike	As^III^, As^V^, MMA, **DMA**, AsB, TMAO, AsC, TETRA, unknown	70.5±8.1	Zheng and Hintelmann, 2004
	Large mouth bass	As^III^, As^V^, DMA, AsB, TMAO, AsC, **TETRA**, unknown	88.7±7.6	
	Yellow perch	As^III^, **As**^**V**^, MMA, DMA, AsB, TMAO, AsC, TETRA, unknown	77.8±7.2	
	Pumpkinseed	As^III^, As^V^, MMA, DMA, AsB, TMAO, AsC, **TETRA**, unknown	66.8±5.9	
Methanol:water 9:1, shaken overnight at room temp, and centrifuged for 15 min at 3000 r/min; Second extraction: 10 mL methanol/water, shaken 30 min, and evaporated	Catfish	**AsB**	0.4	Šlejkovec *et al.*, 2004
	Burbot	**DMA**, AsB, unknown	77.2	
	Barbel	**AsB**, unknown	18.5	
	Danube roach 1	**AsB**, unknown	36.2	
	Danube roach 2	TMAO, **AsB**, unknown	25.9	
	Nase 1	DMA, TMAO, **unknown**	7.6	
	Nase 2	**DMA**, AsB, unknown	7.1	
	Nase 3	**DMA**	2.4	
	Marble trout	**AsB**	90.4	
	Rainbow trout 1, 2	**AsB**	79.6, 74.9	
	Brown trout 1	**AsB**	60.1	
	Brown trout 2	**AsB**, As^III^	73.9	
	Brown trout 3, 4	DMA, **AsB**	70.3, 53.5	
Pure water, MAE: 800 W, 15 min to 80 °C, 15 min at 80 °C. Evaporated	Sliver bream	**AsB**	89	Komorowicz *et al.*, 2019
	Bream	**AsB**	98	
	Carp	**AsB**	94	
	Trout	**AsB**, As^V^	93	
	Sturgeon	**AsB**, As^V^	93	
Pure water, MAE: 30 min at 80 °C. Evaporated	Eel	**AsB**, DMA, MMA	≈70	Ruttens *et al.*, 2012
	Perch	**AsB**, MMA	≈110	
	Pike–perch	**AsB**, DMA	46	
	Trout	**AsB**, DMA	≈72	
	Crayfish	**AsB**, DMA	37	
1% HCl and 0.1% protease XIV. MAE: 1 h at 70 °C	Freshwater tilapia	**AsB**, As^V^, DMA, MMA, As^III^	96	Chen and Jiang, 2021
	Freshwater bass	**AsB**, As^V^, DMA, MMA, As^III^	95	

The arsenic species in bold font are the predominant arsenic species in the fish sample analyzed.

## Separation of Arsenic Species

Hundreds of arsenic species are present in the environment and biological systems (Cullen and Reimer, 1989; Francesconi, 2010; Shen *et al.*, 2013; Reid *et al.*, 2020; Xue *et al.*, 2022), which highlights the need for efficient separation. High-performance liquid chromatography (HPLC) and capillary electrophoresis (CE) are suitable for the separation of arsenic species in liquid samples, such as extracts of fish.

CE has a very high separation efficiency and is capable of separating neutral, anionic, and cationic species simultaneously. However, for the following three main reasons, CE has not been widely accepted for arsenic speciation analysis in environmental and food samples. First, coupling CE separation with inductively coupled plasma mass spectrometry (ICPMS) detection is challenging because the flow rate of separation buffers in CE (µL/min) is much lower than the optimum flow rate of ICPMS sample introduction (typically 1 mL/min). Thus, CE requires a specialized interface to be coupled to ICPMS for sensitive detection (Liu *et al.*, 1995; Holderbeke *et al.*, 1999; Liu *et al.*, 2013). Second, migration time shift between repeated analyses is a common issue in CE (Qu *et al.*, 2015), especially when the sample matrix is complex. Third, the small capillary (10−100 µm internal diameter) limits the sample injection volume to the nL level, which sacrifices the concentration detection limit.

HPLC is more commonly used with ICPMS detection because of the compatible flow rate, lower matrix effect, and better concentration detection limits. A recent review focusing on chromatographic separations of arsenic species described the many different methods used for arsenic species separation in detail (Reid *et al.*, 2020). Briefly described below are several recent studies that involved the separation of arsenic species using common HPLC techniques, including anion exchange, cation exchange, ion pairing, and reverse phase chromatography.

### Anion exchange liquid chromatography

Due to the charged nature of many arsenic species (low pK_a_), anion exchange chromatography has been commonly used for the separation of arsenic species, including As^III^, As^V^, MMA, DMA, AsB, AsC, arsenosugars, and phenylarsenicals (Liu *et al.*, 2018; Reis and Duarte, 2018). Anion exchange columns are usually composed of a silica or polymer backbone with positively charged moieties, typically nitrogen-containing groups such as quaternary amines and amides. Using these types of columns, many studies have reported baseline separation of As^III^, As^V^, MMA, and DMA, which are the most commonly detected species of arsenic. As^III^ often elutes early because it has a higher pK_a_ (9.2) and exists as a neutral species in most mobile phases (pH lower than the pK_a_), resulting in little retention in an anion exchange column. Other non-anionic arsenic species can co-elute with As^III^. This is particularly problematic in the analysis of organisms that contain a high concentration of AsB. The zwitterion AsB can co-elute with the neutral As^III^. Reid *et al.* (2020) discussed different strategies to achieve separation between As^III^ and AsB, as well as other neutral and cationic species. One such strategy used a gradient elution in which mobile phase A was 5% methanol in water and mobile phase B was 5% methanol and 60 mmol/L ammonium bicarbonate buffer with the pH adjusted to 8.7 (Peng *et al.*, 2014; Liu *et al.*, 2015; Yong *et al.*, 2016; Peng *et al.*, 2017). This step gradient resulted in the separation of AsB from As^III^.

### Cation exchange liquid chromatography

Cation exchange chromatography is also commonly used for arsenic speciation analysis, particularly for cationic and neutral species that do not separate on an anion exchange column, such as AsB, AsC, TMAO, TETRA, and some arsenosugars. Cation exchange chromatography works in a similar manner to anion exchange except that the stationary phase is composed of negatively charged compounds, such as sulfate, sulfonate, or carbonate groups. These negatively charged groups interact with the positively charged arsenic species. Xiong *et al.* (2020) showed efficient ­separation of DMA, AsB, TMAO, AsC, and TETRA in the analysis of steamed salmon samples. The cation exchange separation took 16 min under isocratic elution conditions. In a large study on common foods consumed by the German population, Hackethal *et al.* (2021) determined both total arsenic and arsenic species in various types of food. Cation exchange was used to separate AsB from other arsenic species in freshwater and marine fish. These studies, among others, also highlight the challenge of co-elution of AsB and As^III^. The use of gradient elution and other complementary separation techniques are necessary for these two species to be resolved.

### Reverse-phase liquid chromatography

Reverse-phase HPLC (RP-HPLC) is particularly useful for the analysis of arsenolipids (Amayo *et al.*, 2011, 2014; Liu *et al.*, 2021, 2022), which contain fatty acid chains. RP-HPLC has been coupled in parallel to ICPMS and electrospray ionization mass spectrometry (ESIMS) for the determination and identification of many arsenolipids in fish (Amayo *et al.*, 2011, 2014; Arroyo-Abad *et al.*, 2013, 2016). Arroyo-Abad *et al.* (2016) used a linear gradient elution from 100% mobile phase A (0.1% HCOOH in H_2_O) to 100% mobile phase B (0.1% HCOOH in MeOH) to achieve separation between six arsenolipids, AsB, and two unknown arsenic species in 60 min. Xiong *et al.* (2020) used a phenyl-hexyl column to separate arsenic-containing hydrocarbons (AsHC). They achieved baseline resolution between AsHC 404 and AsHC 360 and partial separation from AsHC 332. Yehiayan *et al.* (2009) used a Spherisorb C8 column with a gradient elution using 0.1% formic acid and acetonitrile to separate four As–glutathione conjugates.

### Ion pair liquid chromatography

An advantage of ion pair chromatography is that both ionic and neutral species can be separated. Ion pair chromatography uses a standard reverse-phase column, e.g. C18 or C8, and ion pair reagents, e.g. alkylsulfonate or tetraalkylammonium, in the mobile phase. These ion pair reagents contain a charged moiety and a hydrophobic region, which interact with the analytes and the stationary phase, respectively. Common ion pair reagents are tetrabutylammonium and tetraethylammonium for the separation of anionic arsenic species and hexanesulfonic acid and 1-pentanesulfonic acid for the separation of cationic arsenic species. Organic modifiers are typically added to the mobile phase to decrease retention time and modify selectivity; methanol and acetonitrile are two commonly used organic modifiers. Chen and Jiang (2021) achieved separation of AsB, As^III^, MMA, DMA, and As^V^ in 4.5 min using a C18 column with 1-octanesulfonate as the ion-pair reagent. The authors successfully applied the method to speciation analysis of arsenic in freshwater and marine fish samples. Shi *et al.* (2019) used an enhanced C18 column with a mobile phase consisting of 20 mmol/L citric acid and 5 mmol/L sodium hexanesulfonate adjusted to a pH of 4.3 to achieve separation of As^III^, As^V^, MMA, and DMA in under 4 min.

A careful combination of anion and cation ion pair reagents can be used to simultaneously separate anion, cation, and neutral species. Morita *et al.* (2007) developed a mixed ion pair method using a combination of sodium butanesulfonate and tetramethylammonium hydroxide as the ion pairing reagent to separate As^V^, As^III^, MMA, DMA, AsB, TMAO, TETRA, and AsC in under 12 min. Nan *et al.* (2018) used a similar method to separate As^V^, As^III^, DMA, AsB, TMAO, TETRA, and three unknown arsenic species in under 15 min.

### Multiple modes of high-performance liquid chromatography separation

Most arsenic speciation studies use a single chromatographic technique for separation followed by specific detection of the arsenic species. Detection techniques, such as ICPMS, cannot differentiate the co-eluting arsenic species. In applications where all possible arsenic species are to be analyzed, multiple separation modes may be required. Successful examples include multiple analyses of the same sample using complementary chromatographic separation (Soeroes *et al.*, 2005a, 2005b; Schaeffer *et al.*, 2006; Chen *et al.*, 2010; Molin *et al.*, 2012). Molin *et al.* (2012) showed the sequential use of anion, cation, and reverse-phase chromatography to measure 17 arsenic species in urine. The combination of anion and cation exchange columns was successfully used to separate arsenic species in freshwater organisms (Soeroes *et al.*, 2005a, 2005b; Schaeffer *et al.*, 2006). The combined use of anion and cation exchange columns followed by ICPMS detection enabled the detection of 14 arsenic species.

## Detection of Arsenic Species

In principle, any spectrometric detector capable of element specific detection can be used to detect arsenic. The most commonly applied techniques are atomic absorption spectrometry (AAS), atomic fluorescence spectrometry (AFS), and mass spectrometry (MS) (Gong *et al.*, 2002; Luvonga *et al.*, 2020). AAS and AFS are affordable, and coupling with hydride generation (HG) allows for sensitive detection of arsenic. Adjusting hydride generation conditions could also allow for selective detection of trivalent and pentavalent arsenic species (Matoušek *et al.*, 2013). While the use of HG enhances sensitivity by isolating arsenic species from the sample matrix and dramatically decreases potential spectroscopic interferences, HG is limited to arsenic species that can produce hydrides (Luvonga *et al.*, 2020; Wu *et al.*, 2022). Moreover, a study by Regmi *et al.* (2007) suggests that thiolated arsenicals behave similarly to trivalent arsenic compounds and could cause potential misidentification. The authors also showed that arsenosugars can be volatilized through the breakage of the As–C bond with the longer carbon chain containing a riboside moiety (Regmi *et al.*, 2007).

A wide range of arsenic species can be detected using inductively coupled plasma (ICP) atomic (optical) emission spectrometry (AES or OES). However, ICPAES does not provide sufficient sensitivity for low concentrations of arsenic species. High sensitivity and detection of all arsenic species can be achieved with ICPMS.

### Inductively coupled plasma mass spectrometry detection

ICPMS is one of the most widely used techniques for the detection of arsenic species in the past four decades (Beauchemin *et al.*, 1988, 1989; Feldmann *et al.*, 1994; Day *et al.*, 2002; Currier *et al.*, 2013; Pereira *et al.*, 2016; Luvonga *et al.*, 2020, Lescord *et al.*, 2022). For arsenic speciation, ICPMS is ­commonly coupled with HPLC separation because HPLC and ICPMS have good compatibility in flow-through mode and their combination takes advantage of efficient separation and highly sensitive detection.

ICP can be destabilized by high amounts of organic solvents (Lajin and Goessler, 2021); therefore, coupling ICP plasma with reversed-phase HPLC requires adjustments in experimental conditions. Introduction of oxygen gas to the plasma and post column dilutions were effective methods to increase ICP tolerance to high organic content mobile phases. However, these approaches result in added complexity and decreased sensitivity. Recent research has suggested several alternative eluents to mobile phases with a high percentage of organic solvent. Dimethylcarbonate was tested as an alternative eluent due to its higher elution strength compared to acetonitrile or methanol, but its concentration was limited to <10% by volume and did not allow elution of highly hydrophobic species (Lajin and Goessler, 2021). Lajin *et al.* (2022) proposed the use of 1,2-hexanediol as an eluent that provides both high elution strength and low carbon load. They demonstrated very low detection limits (0.003 μg As/L) for arsenic fatty acids at 10% 1,2-hexanediol in the mobile phase under a standard ICPMS set up.

ICPMS detection is not free of interference. Arsenic is a monoisotopic element and, normally, As^+^ ions are monitored at a mass-to-charge ratio (*m*/*z*) of 75; however, the presence of chloride in samples can interfere with arsenic detection due to the formation of polyatomic ions, such as ^40^Ar^35^Cl^+^ (*m*/*z* 75), in argon plasma. To observe the presence of interference, Soeroes *et al.* (2005a, 2005b) monitored *m*/*z* 77 (^40^Ar^37^Cl^+^, ^77^Se) and *m*/*z* 82 (^82^Se). This ^40^Ar^37^Cl^+^ interference can be estimated based on the natural ratio of chloride isotopes, ^35^Cl:^37^Cl=3:1.

A common method used to reduce interference, particularly polyatomic interference, is to introduce a collision or reaction gas into the collision cell. The sample ions are sent through this compartment before moving into the mass analyzer. This reduces interferences via kinetic energy discrimination. Because polyatomic ions, such as ArCl^+^, have larger cross-sectional areas than As^+^ ions, they undergo more collisions with the collision gas. As a result, the polyatomic ions lose more kinetic energy and are unable to reach the detector. Jones *et al.* (2021) tested the accuracy of arsenic determination under three modes: (1) kinetic energy discrimination (KED) with He as a collision gas; (2) dynamic reaction cell (DRC) with 10% H_2_ and 90% argon; and (3) DRC mode with O_2_ as a reaction gas. All investigated gases enabled similar accuracy. In addition, NH_3_ and CH_4_ have also been used as collision/reaction gases to lessen the effects from polyatomic species; however, their ability to react with arsenic needs to be taken into consideration (Tanner *et al.*, 2002; Grotti and Frache, 2007; Jones *et al.*, 2021).

Oxygen as a reaction gas is introduced into the collision/reaction cell to convert As^+^ (*m*/*z* 75) to AsO^+^ (*m*/*z* 91). Monitoring *m*/*z* 91 for AsO^+^ overcomes the interference otherwise caused by ArCl^+^ (*m*/*z* 75) (Grotti and Frache, 2007). Miyashita *et al.* (2009) developed an HPLC–ICPMS method and were able to detect 11 arsenic species with limits of quantitation ranging from 0.25 to 0.49 μg/L. Chen and Jiang (2021) reported much lower background for AsO^+^ at *m*/*z* 91 than for As^+^ at *m*/*z* 75 under the same conditions. Using a C18 column for separation and ICPMS detection with oxygen as a reactive gas in a dynamic reaction cell, they achieved limits of detection in the range of 0.005–0.007 μg/L for MMA, DMA, As^III^, As^V^, and AsB.

Chromatography separation prior to ICPMS detection also reduces chloride interference in the determination of arsenic species. For example, ion-exchange chromatography can separate Cl^–^ from arsenic species and reduce the possibility of ArCl^+^ interfering with the detection of arsenic species (Sheppard *et al.*, 1990).

Another potential source of interference in arsenic determination is doubly charged ions. Isotopes of rare earth elements ^150^Nd and ^150^Sm can form doubly charged ions in argon plasma with *m*/*z* 75. Single quadrupole instruments with a helium collision cell or an H_2_ reaction cell could not effectively reduce the interference from the doubly charged ^150^Nd^2+^ and ^150^Sm^2+^. Jackson *et al.* (2015) eliminated both chloride and doubly charged ion interferences while maintaining extremely low detection limits (0.001 μg/L for arsenic) using a triple quadrupole mass spectrometer (QqQ). Such efficient performance was realized in ‘mass shift’ mode, in which ions were filtered at *m*/*z* 75 in Q_1_, and then the transmitted ions reacted with O_2_ in the reaction cell (Q_2_) to form AsO^+^, which was followed by mass filtration at *m*/*z* 91 in Q_3_. Other studies have successfully applied ICP-QqQMS to the analysis of arsenic in nursing mothers’ milk (Xiong *et al.*, 2020) and environmental water samples (Stetson *et al.*, 2021).

Unknown arsenic species in multiple freshwater fish samples have been separated using HPLC and detected using ICPMS (Šlejkovec *et al.*, 2004; Zheng and Hintelmann, 2004), such as in freshwater fish *Rhinogobius* sp. (Miyashita *et al.*, 2009) and in migratory *Salmo salar* (Xiong *et al.*, 2020). The identification of unknown arsenic species requires accurate molecular mass and fragmentation pattern information, which cannot be obtained from ICPMS detection. Complementary techniques, such as electrospray ionization mass spectrometry (ESIMS), X-ray absorption near edge structure (XANES), and nuclear magnetic resonance (NMR), need to be used to obtain molecular and structural information. Due to the lower sensitivity of XANES and NMR techniques, ESIMS has been the preferred method in the investigation of unknown arsenic species present at trace concentrations.

### Electrospray ionization mass spectrometry detection

Electrospray ionization (ESI) is a ‘soft’ ionization technique, meaning that the molecular ions are usually maintained during the ionization process, as opposed to the formation of elemental ions in ICP. ESIMS is advantageous for species identification because it provides molecular information on the detected species. Its main disadvantage is poorer detection limits caused by low ionization efficiencies of some arsenic species and ion suppression from the sample matrix (Feldmann, 2005).

Efficient ionization depends on several parameters, such as the complexity of the sample matrix, solvent composition, solution pH, flow rate, voltage, temperature, and gas flow. The formation of gas-phase ions depends on the proportion of the ionized form in solution, which in turn depends on the compound pK_a_ and solution pH. Positively charged ions (e.g. AsB and AsC) are efficiently ionized in positive mode (Cullen *et al.*, 2016). Positive mode allows for effective ionization of arsenolipids (Liu *et al.*, 2021, 2022; Taylor and Karagas, 2022) and many arsenosugars (Miguens-Rodriguez *et al.*, 2002; Lorenc *et al.*, 2020). Pergantis *et al.* (2000) suggested that negative mode allowed for more informative fragmentation spectra of dimethylarsionylribosides, while trimethylarsonioribosides were efficiently characterized in positive mode. Miguens-Rodriguez *et al.* (2002) observed higher signal intensities of arsenosugars in the positive mode despite using anion-exchange separation, implying that arsenosugars were negatively charged in the liquid phase. This phenomenon has been referred to as ‘wrong-way round ionization’.

The use of highly aqueous mobile phases in anion and cation exchange chromatography for the separation of arsenic species presents a challenge for ESI. Because water has relatively high surface tension, highly aqueous mobile phases complicate ion transfer from liquid droplets to the gas phase. To avoid poor ion transfer and ionization efficiency, researchers often switch to reversed-phase HPLC with mobile phases containing higher concentrations of organic solvents. Ionization efficiency can be increased to an extent by increasing the content of organic modifier (e.g. methanol) and optimizing ion source parameters (in particular, temperature and drying gas flow). A higher percentage of organic solvent decreases the surface tension of the droplets in electrospray and increases the efficiency of ion transfer from the liquid droplet to the gas phase. The effect of organic solvent content may differ for different arsenic species as it depends on the solvation energies of specific ions (Corr and Larsen, 1996).

Another key aspect of efficient ionization is potential ion suppression by complex matrices. Sample pretreatment (clean-up) allows for the isolation of arsenic compounds of interest from major interferences. Thus, severe ion suppression can be avoided, and the detection limit can be improved by lowering the matrix-induced background (Domínguez-Álvarez, 2020).

ESIMS has been successfully used for the identification of new arsenic species (Lorenc *et al.*, 2020). ESI is readily combined with a variety of mass analyzers, such as ion trap (IT), QqQ, time of flight (TOF), Orbitrap, and Fourier-transform ion cyclotron resonance (FT-ICR). Mass spectra of both the molecular ion and fragment ions provide information on the identity of arsenic species.

Accurate molecular mass, measured using high-resolution mass spectrometers, serves as a basis for determining the molecular formulas of unknown species. High-resolution mass spectrometers can also provide an additional separation dimension based on the mass-to-charge ratio (*m*/*z*), allowing co-eluting compounds to be differentiated. Commonly used high mass accuracy analyzers include TOF (mass accuracy at low ppm level), Orbitrap (sub-ppm), and FT-ICR (sub-ppm) (Luvonga *et al.*, 2020).

Tandem MS (MS/MS) provides information on characteristic fragment ions and assists in the identification of unknown compounds through matching MS/MS spectra. In tandem MS, the common scan modes of choice are selected reaction monitoring (SRM) or multiple reaction monitoring (MRM), which have the additional benefits of increased sensitivity and selectivity.

Apart from the optimized MS hardware, developments in software enable more coverage of unknown organoarsenicals in complex biological and environmental matrices. Liu *et al.* (2021) developed the data processing software *Precursorfinder*, which enables non-targeted screening of arsenolipids based on characteristic fragment ions: As(CH_3_)^2+^, As(CH_2_)^+^, and As(CH_3_)_2_OH^2+^. Using HPLC-ESIqTOFMS with data-independent acquisition, the authors identified 23 arsenolipids in four types of marine food samples (Liu *et al.*, 2022). *Precursorfinder* was later applied using known arsenolipids fragments As(CH_3_)_2_ and As(CH_2_)_2_. The method has also enabled the identification of AsB, TMAO, and thiolated trimethylarsinic acid. Taylor and Karagas (2022) successfully used precursor ion scan (monitoring ions at *m*/*z* 123, 119, and 105) for the identification of arsenolipids.

Another challenge is associated with the structural characterization of arsenolipids with unsaturated carbon–­carbon (C–C) bonds. Coniglio *et al.* (2022) addressed this challenge by using reversed-phase HPLC, epoxidation reaction, and tandem MS (MS^3^) for structural characterization. The method was successfully applied to establish a double C-C bond position in phospholipids extracted from algae. The authors noted that the method was not able to differentiate *trans*- from *cis*- isomers.

### Simultaneous detection with inductively coupled plasma mass spectrometry and electrospray ionization mass spectrometry

For speciation, ESIMS is best used simultaneously with ICPMS after HPLC separation with a split flow between the two detectors. Simultaneous detection of the same HPLC effluent with ICPMS and ESIMS is more beneficial than two separate analyses, HPLC-ICPMS and HPLC-ESIMS. One reason for this is the possibility of retention time fluctuations caused by matrix effects and inherent uncertainties associated with analytical methods. The advantages of obtaining both elemental and molecular mass information allowed Peng *et al.* (2014) to prevent misidentification of a Roxarsone metabolite, *N*-acetyl-4-hydroxyphenylarsonic acid (*N*-AHPAA) which had an identical retention time to 4-amino-phenylarsonic acid (4-APAA). Another advantage of the simultaneous ICPMS/ESIMS detection technique is the identification of a wide range of arsenic compounds without the need for standards which are often unavailable for complex organoarsenicals. Parallel use of ESIMS and ICPMS allowed the identification of eight known organoarsenicals in marine fish oil (Pereira *et al.*, 2016). The authors were able to identify and characterize five new arsenic-containing fatty acids based on characteristic ions in MS/MS spectra. Further analysis is needed to determine the position of the double bonds in unsaturated arsenic-containing fatty acids.

For simultaneous ICPMS and ESIMS detection, it is important to optimize the mobile phase that enables efficient HPLC separation and is compatible with both ionization techniques. For example, methanol and acetonitrile are common in mobile phases for reversed-phase chromatography, and are suitable for ESI. However, high concentrations of methanol and ­­acetonitrile can cause ICP instability, and their incomplete combustion in ICP may result in a clogged sampling orifice and decreased sensitivity. Multiple approaches have been applied to overcome incompatibility issues including the addition of oxygen to ICP to reduce carbon build up, post-column dilution of organic mobile phases, and lowering the flow rate of organic mobile phases (Luvonga *et al.*, 2020). Appropriate HPLC separation with compatible mobile phases facilitates ICPMS and ESIMS to provide complementary elemental and molecular information for arsenic species identification and quantification.

Complementary use of ESIMS and ICPMS, along with HPLC separation, has enabled the identification of a variety of arsenic species in different sample matrices (Arroyo-Abad *et al.*, 2016; Pereira *et al.*, 2016; Viczek *et al.*, 2016; Peng, *et al.*, 2017; Lorenc *et al.*, 2020; Liu *et al.*, 2021). Using HPLC separation with parallel ESIqTOFMS and ICPMS analysis, Arroyo-Abad *et al.* (2016) identified four arsenic fatty acids (AsFA) and two arsenic-containing hydrocarbons (AsHC) in freshwater fish. Hyphenated techniques allowed the identification of a wide range of organoarsenicals in seafood, including a new group of arsenolipids, phosphatidylcholines (Viczek *et al.*, 2016). Twelve arsenosugars were identified in algae samples using HPLC-ICPMS in combination with ultra-high-performance liquid chromatography coupled to electrospray ionization high-resolution mass spectrometry (UHPLC-ESIHRMS) (Lorenc *et al.*, 2020).

## Concluding Remarks

Food consumption is one of the main routes of human exposure to arsenic. Various arsenic species present in food have very different chemical properties and toxicities. Therefore, studies of arsenic speciation in food are critical for any meaningful assessment of food quality. A wide range of arsenic species have been reported to be present in freshwater fish, although their concentrations vary greatly depending on the fish species, habitat, and water arsenic concentration, among many biological and environmental factors. Further research is needed to identify arsenic species in freshwater fish and characterize their chemical and toxicological properties.

The identification, characterization, and quantification of arsenic species in food, including freshwater fish, require multiple analytical techniques. HPLC separation in combination with ESIMS and ICPMS detection provides ­complementary chemical information on arsenic species, including their ­chromatographic retention times, *m*/*z* of molecular and ­fragment ions, and quantitative amounts. The complementary information is useful for the quantitative determination of individual arsenic species. Further research on improving separation efficiency, identification ability, and detection sensitivity will help characterize currently ‘unknown’ arsenic species.

Arsenic species in solid food samples must be extracted into liquid solution for subsequent analysis using HPLC-ICPMS and HPLC-ESIMS. The method of extraction must maintain the integrity and stability of arsenic species. Due to differences in the nature of food samples, even among different fish samples, extraction efficiencies vary with different types of food, even with different fish tissues. Quantitative extraction of arsenic species while preserving their chemical identify is crucial for the quantitative determination of arsenic species in food. Extractions with different solvents and treatment with appropriate enzymes will help improve the extraction efficiency.

There is a shortage of sensitive techniques for direct determination of arsenic species in food materials without sample treatment. Most available direct analysis techniques do not have the sensitivity and specificity necessary for the determination of diverse arsenic species that may be present at trace concentrations. The developments of new techniques and methods for direct determination of arsenic species in food will benefit studies of food quality and safety pertaining to arsenic.
